# Unusual presentation of a first branchial arch fistula with maxillofacial infection: a case report

**DOI:** 10.1186/s12893-021-01303-2

**Published:** 2021-07-03

**Authors:** Yu Han, Run-qin Yang, Liu Hong, Cui-ping Zhong, Ding-jun Zha

**Affiliations:** 1 Department of Otolaryngology, Xijing Hospital, Fourth Military Medical University, Xi’an, 710032 Shaanxi Province China; 2 State Key Laboratory of Cancer Biology, National Clinical Research Center for Digestive Diseases, and Xijing Hospital of Digestive Diseases, Fourth Military Medical University, Xi’an, 710032 Shaanxi Province China; 3 Department of Otolaryngology Head and Neck Surgery, the 940Th Hospital, Lanzhou, 730050 Gansu Province China

**Keywords:** First branchial cleft anomalies, Facial nerve, Maxillofacial, Case report

## Abstract

**Background:**

First branchial cleft anomaly (FBCA) is a rare congenital defect that arises due to incomplete closure of the ventral portion of the first and second branchial arches. There are variable complex clinical manifestations for patients with FBCA, which are prone to misdiagnosis and inadequate treatment. FBCAs usually involve the facial nerve with a consequent increased risk of facial nerve damage. Here, we present an unusual case of FBCA presenting with two preauricular pits in association with an abnormal maxillofacial cyst.

**Case presentation:**

A 10-month-old girl presented to our department due to recurrent maxillofacial infections accompanied by swelling or abscess of the left cheek and purulent discharge from the preauricular pit for 4 months. A 3D-computed tomography (CT) fistulogram and magnetic resonance imaging (MRI) revealed two conjunctive tract lesions: one tract arose from the skin surface anteroinferior to the external auditory canal (EAC), through the deep lobe of the left parotid, and anteriorly extended to the left masseter; the other extended from the superficial lobe of the left parotid to the intertragic notch. After the maxillofacial infection was controlled by intravenous antibiotic administration, surgery was performed. Intraoperative tools, such as facial nerve monitors, microscopes, and methylene blue dyes, were used to facilitate the complete dissection and protection of the facial nerve. On follow-up over one year, the patient recovered well without facial palsy or recurrence.

**Conclusion:**

FBCA with maxillofacial cysts is rare and prone to misdiagnosis. Physicians should pay attention to this anatomic variant of FBCA with the fistula track located deep inside the facial nerve and projected medially to the masseter.

## Background

First branchial cleft anomalies (FBCAs) are rare congenital defects that account for less than 8–10% of all branchial cleft anomalies and arise due to incomplete closure of the ventral portion of the first and second branchial arches [1, 2]. A number of classification systems have been developed in an attempt to assist preoperative assessment and surgical planning for FBCAs. Work’s classification is the most common system based upon both anatomical and histological features. According to this classification, FBCAs are divided into two distinct types: type I has only ectodermal components and is usually superficial to the facial nerve and lies in close proximity to the ear; type II has ectodermal and mesodermal components, often lying medial to the facial nerve and communicating with the external auditory canal (EAC) [3]. Olsen et al. [4] also introduced a classification of defects into cysts, sinuses, or fistulas based on the number of surface openings present. To determine the relationship between FBCAs and the facial nerve prior to surgery, Liu et al. introduced a new subclassification for type II FBCAs into three subtypes based on MRI findings [2]. A common location of FBCAs is the area from the EAC to the level of the hyoid bone. The opening of the fistula of FBCAs is typically located in the periauricular area, but more rare locations have also been reported, such as the EAC, middle ear cleft, postauricular region, and even within the neck over the angle of the mandible [5]. Usually, FBCAs have a close anatomical relationship to the facial nerve owing to their embryologic origin. D’Souza et al.[6] performed a comprehensive review of the literature and found that FBCAs had diversiform patterns with the facial nerve; they can be lateral to, medial to, or between branches of the facial nerve. Patients presenting at a younger age were more likely to have a deep tract with a consequent increased risk of facial nerve damage. Thus, treatment for FBCAs is complicated by both variable tract lesions and the complex anatomical relationship to the facial nerve. In this article, we describe a paediatric case of an exceptional type II FBCA presenting with two preauricular pits in association with an abnormal maxillofacial cyst and a tract that passes into the deep lobe of the parotid gland and extends anteriorly to the masseter.

## Case presentation

**Fig. 1:**
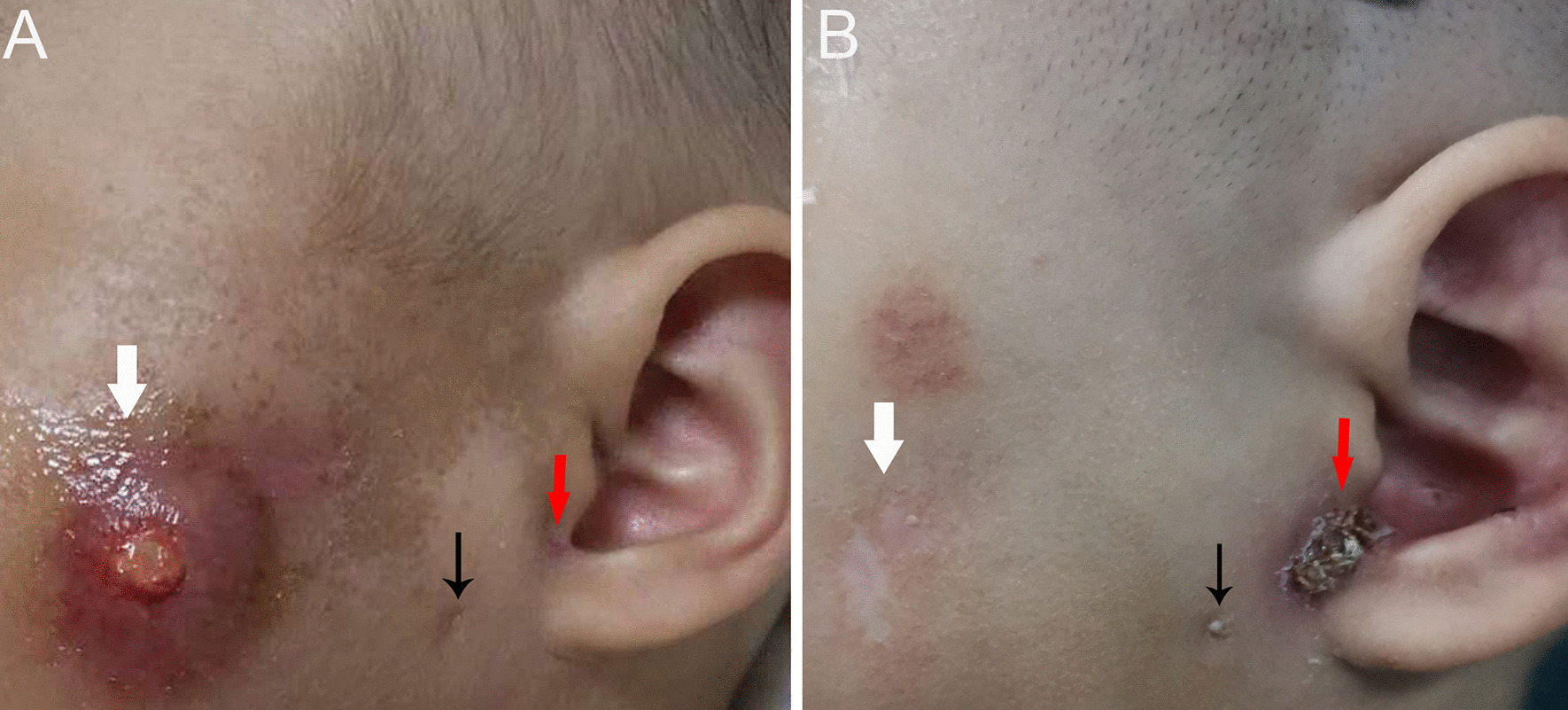
First branchial cleft anomalies presenting as two preauricular pits associated with an abnormal maxillofacial cyst. One pit with purulent secretions is in the intertragic notch (red arrow), and another small cutaneous dimple (black arrow) is shown in the left cheek region. The maxillofacial infection with abscess resolved with conservative treatment. **A** The black arrow indicates the cutaneous dimple before treatment, and **B** the black arrow shows the same region post-treatment

**Fig. 2:**
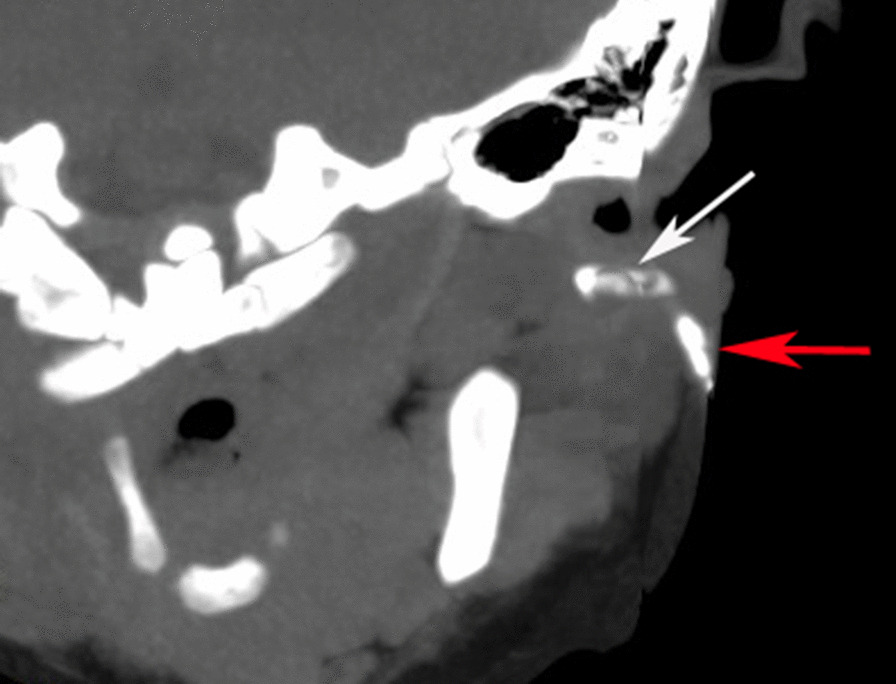
3D-coronal computed tomography fistulogram scans are shown. Low osmolar nonionic iodinated contrast was injected through the intertragic notch dimple, revealing two tracts, one extending anteriorly to the left masseter (white arrow) and another lying in the superficial lobe of the left parotid (red arrow)

**Fig. 3:**
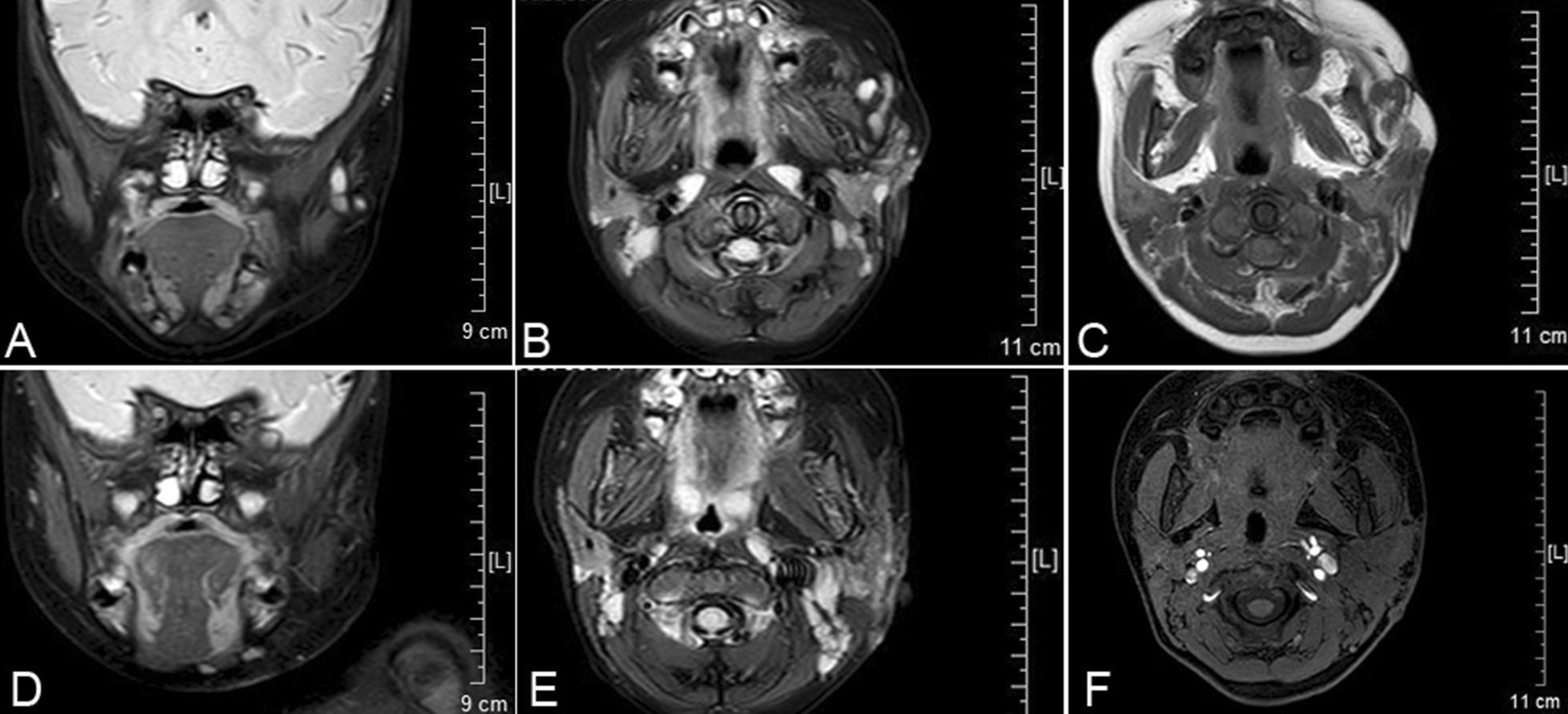
Magnetic resonance imaging (MRI) is shown. Before surgery treatment, coronal T2 weighted MRI (**A**), axial T2 weighted MRI (**B**) and axial T1 weighted MRI (**C**) reveal the location of a mass centred between the masseter muscle and left parotid gland. At follow-up over one year postoperatively, coronal T2 weighted image (**D**), axial T2 weighted image (**E**) and 3D mDIXON-axial fat suppressed T1 weighted image (**F**) confirm there is no recurrence on the primary site of the lesion

We obtained written informed consent from the patient’s parents. The case is compliant to the SCARE guidelines [7]. A 10-month-old girl was taken to our hospital by her parents due to recurrent maxillofacial infections accompanied by swelling or abscess of the left cheek and purulent discharge from the preauricular pit for 4 months. On physical examination, a swollen erythematous maxillofacial lesion was observed in the left cheek (Fig. 1). A pit with white purulent secretions in the intertragic notch and another small cutaneous dimple in the left parotid gland region were observed (Fig. 1A). EAC contained no fistula track. All other head and neck examinations were unremarkable. Ultrasound performed for the maxillofacial area demonstrated a lesion with inflammatory changes. The other auxiliary examinations, including otoscopy, pure tone audiometry and renal ultrasound, were without abnormalities. There was no history of previous incision or drainage procedures. No particular family history was recorded. A 3D-computed tomography (CT) fistulogram and magnetic resonance imaging (MRI) were performed to delineate the course of the tract and the extent of the lesion. The results revealed two conjunctive tracts: one tract arose from the skin surface anteroinferior to the EAC, beneath the facial nerve, and passed into the deep lobe of the parotid gland and projected to the masseter; the other tract extended from the superficial lobe of the left parotid to the intertragic notch (Figs. 2 and 3A–C).

**Fig. 4:**
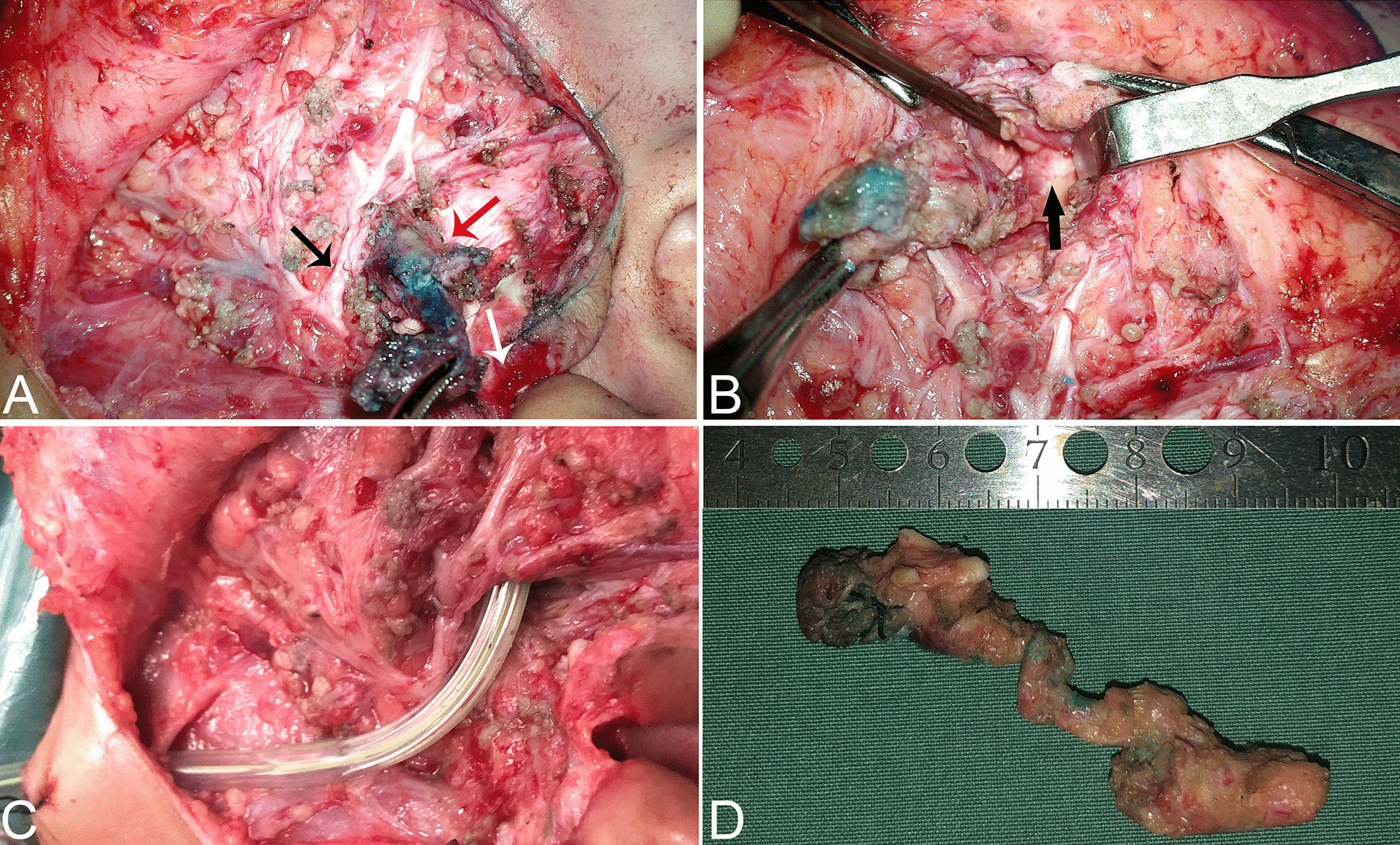
Intraoperative findings and surgical strategy. **A** The facial nerve was identified during surgery. The larger tract (red arrow) projecting medially deep within the temporal trunk of the facial nerve (black arrow) and the smaller tract ending at the cartilage of the intertragic notch (white arrow) are shown. **B** The black arrow indicates the lesion boundary as a blind pouch lying tightly along the masseter. **C** A suction drain was placed into the tract tunnel after the first branchial cleft anomaly excision. **D** A specimen containing cartilage post-removal is shown. The complete fistula was approximately 6 cm in length

Originally, the infection could be controlled by intravenous antibiotic administration, which resulted in maxillofacial infection improvement (Fig. 1B). However, recurrent inflammation had been persisting for one year. Finally, after obtaining parental consent, the patient underwent surgical management under general anaesthesia at 2 years old. The surgery was performed under facial nerve monitoring. First, a methylene blue staining agent was injected into the cutaneous pit of the intertragic notch. Then, a modified Blair incision encompassing the pit was made. The facial nerve was identified first, which allowed the cyst to be incised easily. During superficial parotidectomy, one cyst lying in the superficial lobe of the parotid and cartilage of the intertragic notch were removed. Blunt dissection was then used to follow the other larger fistula tract, which was located deep inside the facial nerve and projected medially to the posterior aspect of the masseter (Fig. 4A). Using a microscope, the tract was carefully separated from the facial nerve, which formed a blind pouch alongside the masseter (Fig. 4B). Finally, a suction drain was placed and kept until 48 h postoperatively (Fig. 4C). The patient recovered well, and no complications occurred in the following days. The final pathology of the excised tissue was consistent with that of type II FBCA (Fig. 4D). At follow-up over one year postoperatively, the surgical site was well healed, and the patient had no facial palsy or recurrence by coronal and axial MRI imaging (Fig. 3D–F).

## Discussion and conclusions

Branchial anomalies arise when the branchial arches and their associated clefts or pouches fail to regress or develop normally [8]. FBCAs are relatively uncommon, and there are various clinical manifestations in patients. As shown in Table 1, FBCAs can appear at any age, and the tract may extend to the osteocartilaginous junction of the ear canal, Eustachian tube, submandibular gland, pharyngeal cavity, and digastric muscle [1, 9–14]. Muranishi et al. [15] also reported a case of FBCA that was clinically typical but occult in images and pathology. In our case, repetitive inflammation around the maxillofacial region was observed. The unusual cord structure beneath the facial nerve, which passed into the deep lobe of the parotid gland, projected to the masseter. This rare case of type II FBCA is prone to misdiagnosis and inadequate treatment. Although this patient had two preauricular pits, the lesion was distinct from congenital preauricular fistula, which is more common and arise from failure of fusion of the auricular hillocks of His instead of the branchial arches [16]. In addition, some rare syndrome diseases, such as Branchio-oto-renal syndrome (BORS) or Goldenhar syndrome, may be accompanied by branchial cleft anomalies. However, these diseases are usually autosomal dominant disorders with abnormalities in multiple organ systems [17, 18]. For example, the typical manifestations of BORS are hearing loss, abnormal branchial cleft development, and renal dysplasia, while patients with Goldenhar syndrome usually present with the presence of congenital cholesteatoma, branchial cleft anomalies and facial nerve abnormalities. Although FBCAs can exhibit different patterns, they are rarely associated with other facial malformations [19]. However, errors in the development of the first branchial cleft can result in duplication anomalies of the external auditory canal [20]. In a few cases, FBCAs are accompanied by microtia, stenosis or atresia of EAC, and cholesteatoma [21–23]. Therefore, the diagnosis of FBCAs is challenging, and doctors should be aware of possible anatomic variants for this disease and keep in mind the differential diagnosis of some rare syndromes.

**Table 1:** Representative clinical manifestations of FBCA patients

Author	Date	Age	Complaints of presentation	Description the course of FBCAs
Fastenberg [9]	2016	3-year-old	Pain in the right ear and swelling in the postauricular area	A fistula track into the osteocartilaginous junction of the ear canal
Faruque [10]	2019	12-year-old	A draining cervical pit	Fistulas opening to the Eustachian tube
Watanabe [11]	2017	14-month-old	Redness and swelling in the left neck area	Fistula extending from a cutaneous opening in the left submandibular area penetrating the submandibular gland, and ending in the pharyngeal cavity
Fanous [1]	2020	6-year-old	Left conductive hearing loss and an ipsilateral painful cervical mass	A presumed ear canal cholesteatoma in association with an abnormal bony canal and a pharyngeal cyst
Chaouki [12]	2021	3-year-old	Recurrent right lateral cervical infection	A fistulous path underthe facial nerve and ends under the digastric muscle
Roche [13]	2016	4-year-old	Recurrent left neck abscesses and palpable persistent submandibular swelling	Duplication of the external ear canal running medial to the facial nerve
Zhang [14]	2020	19-year-old	A mass behind the right earlobe and recurrent postauricular swelling and pain	The mass originated from the stylomastoid foramen and adhered to the posterior surface of the parotid gland, invading the temporal bone
Muranishi [15]	2020	8-year-old	An infectious epidermal cyst	A cord structure attached to subcutaneous tissue at the intertragal notch, no opening to the external auricular canal

Once FBCA is diagnosed, surgical excision is the definitive treatment choice. However, choosing the optimal surgical time and surgical approach is very important. As reported, recurrence rates were as high as 20% when excision was performed in the acutely inflamed stage [24]. Repeated infection in the lesion might result in inflammatory adhesion in surrounding tissues and cause structures to be difficult to identify, which may increase the risk of recurrence and injury to the facial nerve. Thus, surgical excision should be considered after the infection has resolved. Additionally, age is another factor affecting surgical treatment. As previous studies have reported, the anatomy of infants differs from those of older children and adolescents. The facial nerve of younger children is more superficial and delicate than that of older children and adults, which makes surgical treatment challenging. Furthermore, younger patients are more likely to have lesions deep to the facial nerve, and the facial nerve injury rate ranges from 10 to 25% in paediatric patients undergoing FBCA resection [25]. Thus, we agree with the opinion that surgeons should weigh the risks and benefits of surgery with care when patients are less than 1 year old [26]. Moreover, in most FBCA cases, a parotidectomy approach with preliminary identification of the facial nerve and excision of the entire tract is necessary. Patients with FBCAs often concurrently present with otologic complaints, and they may require ear-specific surgery such as tympanoplasty or canalplasty [27]. Additionally, methylene blue dye injection during surgery may help verify the suspected path of a fistula, and intraoperative microscopy and facial nerve monitoring are indispensable for protecting the facial nerve. These methods have the potential to reduce facial nerve injury and recurrence rates.

In summary, we described an unusual presentation of type II FBCA with maxillofacial infection, and the main fistulous tract travelled medial to the facial nerves into the deep lobe of the parotid gland and extended anteriorly to the masseter. Surgical excision should be performed after the resolution of any infection as it is important for identification of the facial nerve in almost all cases, especially in young patients. Intraoperative tools, such as facial nerve monitors, microscopes, and methylene blue dyes, facilitate the complete dissection and protection of the facial nerve.

## Data Availability

Not applicable.

## Abbreviations

FBCA: First branchial cleft anomaly
FBCAs: First branchial cleft anomalies
EAC: External auditory canal
MRIs: Magnetic resonance images
